# Considerations of psychotic symptomatology in anti‐NMDA encephalitis: Similarity to cycloid psychosis

**DOI:** 10.1002/ccr3.2522

**Published:** 2019-11-07

**Authors:** Eloi Giné Servén, Ester Boix Quintana, Nicolau Guanyabens Buscà, Virginia Casado Ruiz, Cristina Torres Rivas, Marta Niubo Gurgui, Josep Dalmau, Carol Palma

**Affiliations:** ^1^ Hospital de Mataró Consorci Sanitari del Maresme Mataró Spain; ^2^ Hospital Clinic‐IDIBAPS Universitat de Barcelona Barcelona Spain; ^3^ University of Pennsylvania School of Medicine Philadelphia PA USA; ^4^ FPCEE Blanquerna Universitat Ramon‐Llull Barcelona Spain

**Keywords:** anti‐NMDA receptor encephalitis, autoimmune encephalitis, cycloid psychosis, first episode of psychosis, schizophrenia

## Abstract

Most patients with anti‐NMDA receptor (NMDAR) encephalitis present with acute psychosis which is difficult to differentiate from psychotic episodes related to a primarily psychiatric disease. A precise description of the psychiatric phenotype of this disease would greatly facilitate the early diagnosis of these patients. We provide here a detailed description of three of these patients and the similarity of the clinical features with cycloid psychosis. All three patients met Perris and Brockington's criteria for cycloid psychosis in the initial phase of the autoimmune process, including among other an acute and polysymptomatic onset, polymorphous psychotic symptomatology, mood swings, and changes in psychomotricity. In addition, none of the patients had experienced an extended psychiatric prodromal phase. External stress factors preceded symptom onset in the three patients, who also showed common base personality traits and intolerance to a range of antipsychotic treatments. Complementary studies disclosed that the three patients had ovarian teratoma as well as abnormal EEG, and CSF antibodies against NMDAR. Patients with anti‐NMDAR encephalitis may present with clinical features that resemble cycloid psychosis. In addition, our patients did not have prodromal history of psychiatric symptoms and showed intolerance to antipsychotic medication, which all should raise concern for anti‐NMDAR encephalitis, prompting CSF antibody testing.

## Graphical abstract

Clinical features that suggest that a first episode of psychosis is caused by autoimmune encephalitis include the following: (a) lack of long‐term (cognitive and negative) psychiatric prodromes; (b) the presence of an atypical psychotic clinical profile; and (c) hypersensitivity to the side effect of antipsychotic medications.

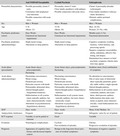

## 1 INTRODUCTION

Approximately 70% of all patients diagnosed with anti‐NMDAR encephalitis exhibit psychiatric symptoms, mainly in the form of acute or subacute onset psychotic episodes characterized by a rapid and serious evolution. Most patients do not have previous history of psychiatric symptoms and are often admitted to psychiatric units. These episodes are usually accompanied by subtle neurological symptoms, which in most patients become more severe during the weeks that follow the initial psychiatric symptoms, including, seizures, abnormal movements, decreased level of consciousness, or dysautonomic features. However, there is a small group of patients who only develop psychosis as manifestation of anti‐NMDAR encephalitis.^1^ Recognition of these patients is important because they also respond to immunotherapy.^2^

Previous studies have described the psychiatric symptoms of patients with anti‐NMDAR encephalitis.^3^, ^4^ These studies are often systematic reviews that list the most frequent abnormal features but do not provide a detailed account regarding the appearance, combination, and evolution of these symptoms. Moreover, although some studies suggest a series of warning signs that can help clinicians to identify anti‐NMDAR encephalitis in patients with psychotic symptoms,^5^, ^6^ many of these signs are based on the identification of clinical neurological features or abnormal tests (eg EEG, CSF). In order to facilitate an early and accurate diagnosis of patients with isolated psychiatric symptoms, it is crucial to focus in a detail clinical description of the psychiatric phenotype of this illness. Here, we report the psychiatric presentation of three patients with anti‐NMDAR encephalitis and discuss the similarity of their symptoms with those in cases of cycloid psychosis.^7^, ^8^

## 2 PATIENT 1

The patient is a 17‐year‐old Caucasian female without previous history of neurological or psychiatric diseases. She displayed adaptive cluster C personality traits (perfectionism and emotional dependency). In June 2011, she had been subjected to an external stress factor connected with the family. She displayed no prodromal psychiatric symptoms, but she did exhibit nonspecific prodromal somatic symptoms (headaches, general discomfort, and high blood pressure).

She presented with acute onset (within 24 hours) of polysymptomatic psychosis, characterized by feelings of strangeness and delusions of self‐reference. Additionally, she showed a high degree of anxiety, distress and confusion, incoherent speech, delusions of guilt, catastrophe and persecutory ideas, and extreme concern with death. She described auditory (noises, imperative voices, and songs) and visual hallucinations (objects and shadows), hypersensitivity to auditory stimuli, and insomnia.

After 72 hours, she was admitted to our Child and Adolescent Psychiatric Unit with an initial diagnostic orientation of an episode of depression with psychotic symptoms. An initial somatic screening including general blood test and head CT scan was normal. For this reason, she was started on fluoxetine 20 mg/day and quetiapine 100 mg/day, which was replaced 3 days later by risperidone 2 mg/day due to features of hypotension (paleness and sedation).

During these first days, the patient showed confusion, disorientation, mood swings (hyporeactivity, irritability, dysphoria, lability, and a feeling of being emotionally overwhelmed) and marked mood fluctuations (euthymia—hypothymia). She experienced changes in thought patterns (disjointed, incoherent, and mental blocks) and in motricity (disorganization, hyperactivity, inhibition, and occasional catatonic posturing). Furthermore, the delusional symptoms, the auditory hallucinations (an expression of perplexity and “listening attitude”), and the global insomnia persisted. At the end of the first week, a number of possible side effects of the psychopharmacological treatment appeared (sedation, a slowing of psychomotricity, and bilateral rigidity). These symptoms evolved toward mutism and catatonia, and some stereotyped movements appeared in the upper extremities, hence complementary examinations were undertaken (see Table 1). Due to the high likelihood of autoimmune encephalitis, the antipsychotic treatment was discontinued and immunosuppressive treatment commenced. In the extension study, an ovarian teratoma was detected and removed. The presence of anti‐NMDA receptor antibodies in the patient's blood and CSF was subsequently confirmed. The patient's psychiatric and cognitive evolution was good, and she was released from the hospital 70 days after she had been admitted.

**Table 1:** Patient characteristics and complementary exploration

Patient, sex, age in years	Premorbid personality	Stressor	Somatic prodromes	Psychiatric prodromes	DUP (hours)	Cycloid Psychosis Criteria	Cranial MRI	EEG	Serum Ac anti‐NMDAR	CSF (num. cells, Ac anti‐NMDAR)	Neoplasia test
#1, Woman, 17	Cluster C traits	Family stressor	Headaches, general discomfort, high blood pressure	No	72 h	Yes	Hyperintensity from both hippocampus and the lower left temporal lobe	Slowing right frontal temporal lobe	Positive	11 cells Positive	Ovarian teratoma
#2, Woman, 23	Cluster C traits	Workplace stressor	No	No	24 h	Yes	Normal	Extreme Delta Brush	Positive	5 cells Positive	Ovarian teratoma
#3 Woman, 35	Cluster C traits	Workplace and economic stressor	No	No	72 h	Yes	Normal	Extreme Delta Brush	Positive	11 cells Positive	Ovarian teratoma

Abbreviation: DUP, Duration of Untreated Psychosis.

## 3 PATIENT 2

The patient is a 23‐year‐old Caucasian woman. She had no history of somatic disorders, and her only psychiatric history was related to her nonadaptive cluster C personality pattern (hyper‐responsible, self‐demanding, and emotionally dependent). In July 2016, in a context of workplace stress, but with no somatic or psychiatric prodromal symptoms, the patient exhibited an acute episode of psychosis that had occurred fully in a 24‐hour period, characterized by suspicion, psycho‐physical anxiety, and a feeling of depersonalization and derealization. Additionally, she showed logorrhea, tachypsychia, and incoherent speech with verbalization of persecutory delusions and self‐reference, and multiple doubts about past events. Plus, she reported hypersensitivity to auditory stimuli, auditory and synaesthetic hallucinations, false recognitions, psychomotor restlessness, and general insomnia.

Upon her arrival, a blood and urine toxic analysis were performed and no alteration was observed. For this reason, she was admitted to the acute care unit for observation with an initial diagnosis impression of brief psychotic disorder. She was prescribed 10 mg/day of olanzapine. In the first days after admission, we observed thymic (hyperthymia—hypothymia) and mood changes (hyperactive and irritable) and abnormal thinking patterns (increased latency, lack of spontaneity, blockages in the course of thought, bradypsychia, and bradyphrenia). She also experienced disinhibition, altered psychomotricity (disorganization, unproductive hyperactivity, inhibition, and slow movements) and cognitive interference (problems with attentional control, short‐term memory). During these days, the patient presented persistence of persecutory delusions and self‐reference, false recognitions and auditory (music and insults) and synaesthetic hallucinations. After 12 days of hospitalization and still no clinical improvement (and even some deterioration in her psychomotricity), the decision was made to proceed with complementary neuroimaging and lumbar puncture studies. The neuroimage and first analytical studies revealed no alterations, but the autoimmune study was still pending. The dose of olanzapine was increased to 20 mg/day. The patient exhibited drowsiness and hypersalivation, so the olanzapine was replaced by 4 mg/day of risperidone on day 18 after admission with the rapid instauration of extrapyramidal side effects. Due to the patient's hypersensitivity to psychopharmacology, a decision was made 20 days after admission to end this treatment and to begin electroconvulsive therapy. Two sessions of this therapy were carried out, which the patient tolerated well, and there was clinical improvement. However, the treatment was interrupted by the results of the test for the anti‐NMDA receptor antibodies in the patient's blood and CSF, which were positive. An extension study discovered an ovarian teratoma, which was removed. Immunosuppressive treatment was initiated, and the response was good. The patient was released 45 days after admission.

## 4 PATIENT 3

This patient is a 35‐year‐old Caucasian woman. She had no somatic medical history of note. In terms of psychiatric history, she had a mild intensity depressive episode 3 years prior. She was treated with antidepressants (20 mg/day of paroxetine) by her primary care physician and exhibited a good clinical response. In July 2017, she was brought to an emergency psychiatric clinic after exhibiting behavioral alterations over a period of 72 hours. The episode occurred at a time when the patient had been experiencing economic and work‐related problems. However, she did not exhibit any psychiatric or somatic prodromes. The initial clinical assessment was characterized by disorganized behavior and intrusive ideas and images and irritability. Additionally, she showed hyperthymia, increased energy, logorrhea, and tachypsychia. Plus, she reported an unusual concern with death, vague fears, and delusional ideas of self‐reference and catastrophe (the belief that something would happen to a family member).

The initial diagnosis orientation was an acute manic episode with mood‐incongruent psychotic features. As such, the patient was prescribed a treatment of up to 20 mg/day of olanzapine and 300 mg/day of valproate, and she was admitted to the hospital's psychiatric unit. Over the first 24‐hour period after her admission, the patient exhibited pronounced clinical fluctuations. She oscillated between periods when she was nearly asymptomatic and others when her symptoms worsened. Those showed persistence of temporal disorientation, auditory hallucinations, false recognitions, and incoherent speech. She verbalized demonic possession, megalomaniac and persecutory ideas, all accompanied by significant behavioral alterations (psychomotor agitation). At the end of the first week after the patient's admission, her clinical presentation had deteriorated, as she was exhibiting hyperthymia, megalomania, logorrhea, incoherent speech, and hallucinatory behavior. In response, a decision was taken to increase the dosage of olanzapine to 30 mg/day, and treatment with 600 mg/day of lithium was also prescribed. The patient showed signs of autonomic dysfunction, low‐grade fever (37.9º), and orofacial movements on the right side of her face, reaching a state of stupor with generalized rigidity in the second week after admission. In light of the strong suspicion that the patient was suffering from autoimmune encephalitis, a complementary extension study was undertaken (see Table 1) and resulted in the discovery of an ovarian teratoma, which was then removed. Treatment with intravenous immunoglobulins and corticotherapy was begun. The patient's evolution was slow, and she displayed severe autonomic dysfunction (urinary retention, severe and persistent fever without a source of localized infection, tachycardia, hypertension, and hypoventilation), requiring orotracheal intubation and a transfer to the intensive care unit. Subsequently, test results confirmed the presence of anti‐AMDAR antibodies in the patient's blood and CSF. Despite the initial treatment, the patient's clinical evolution was poor, and a decision was made to administer weekly treatments with rituximab for a period of a month. A month and a half after her admission, the patient began to display slow and gradual clinical improvement, and she was released from the hospital after 90 days.

## 5 DISCUSSION

In the initial phase of the autoimmune process, our three Anti‐NMDAR encephalitis patients met Perris and Brockington's diagnostic criteria for cycloid psychosis (Table 2). To put in a nutshell: acute and polysymptomatic onset, polymorphous psychotic symptomatology, mood swings, and alterations in psychomotor activity which in all cases evolved into catatonia.

**Table 2:** Perris and Brockington's diagnostic criteria for cycloid psychosis

An acute psychotic episode, unrelated to substance use or to brain organicity, with an onset between 15 and 50 years of age. Sudden onset in a period of hours or of a few days at most. To arrive at a definitive diagnosis, at least four of the following symptoms should be present: Some degree of confusion, ranging from perplexity to severe disorientation. Mood‐incongruent delusions of any kind: most often with a persecutory content. Hallucinatory experiences of any kind, often related to fear of death. An overwhelming, frightening experience of anxiety, not bound to particular situations or circumstances. Deep feelings of happiness or ecstasy, most often of a mystical nature. Akinetic or hyperkinetic motility disturbances. A particular concern with death. Background (oscillations of mood, but not pronounced enough to justify a diagnosis of an affective disorder). There is no fixed symptomatologic combination: on the contrary, the symptomatology may change frequently during the episode

Similarly to what usually occurs in cycloid psychosis, three other factors were observed in our three cases: the absence of long‐term psychiatric prodromes (cognitive and negative symptoms), the presence of external stress factors preceding symptom onset, and cluster C personality traits (including rumination, perfectionism, and emotional dependency). A context of stress has been connected with the onset and exacerbation of autoimmune diseases such as multiple sclerosis 9, 10 and systemic lupus erythematosus.^11^, ^12^ Prior studies have weighed the possibility that certain personality traits are linked to autoimmune diseases.^13^, ^14^, ^15^

Anti‐NMDAR encephalitis, likewise cycloid psychosis, tends to appear most frequently in young women and is usually accompanied by general insomnia and slight mood swings starting a few days before the emergence of symptoms.

Related to antipsychotic medication, anti‐NMDAR encephalitis patients normally show intolerance to antipsychotic medication. They usually display marked extrapyramidal side effects, especially with those medications with greater antagonist affinity for the receptor D2.^16^ This is an unusual trend in cycloid psychosis patients.^17^, ^18^

In anti‐NMDAR encephalitis patients, the psychotic symptoms generally evolve into the appearance of serious neurological complications.^1^ Twenty percent of patients with anti‐NMDAR encephalitis experience subsequent relapses,^19^, ^20^, ^21^ and often in the months following the acute phase, cognitive interference, and clinical issues related to impulse control appear.^22^, ^23^ The previous statements make a major difference from cycloid psychosis. Cycloid psychosis is a clinical entity that most frequently follows a recurring pattern, featuring periods of interepisodic ad integrum recovery marked by an overall lack of residual psychotic symptoms (7%‐17%).^24^, ^25^

Unlike the two clinical entities described above, patients who experience a first episode of psychosis in schizophrenia (Table 3) tend to have a long clinical history of prodromal psychiatric features (negative and cognitive symptoms) that affect their basic functioning.^26^, ^27^ In addition, delusions and hallucinations frequently appear progressively and are latent in these patients, meaning that their psychosis goes untreated for a longer period of time.^28^

**Table 3:** Characteristics of the initial psychotic episode in different entities

	Anti‐NMDAR Encephalitis	Cycloid Psychosis	Schizophrenia
Premorbid characteristics	Possible personality cluster C traits. Correlation with neoplasias/viral infections Possible connection with acute stress.	Personality cluster C traits Close family member(s) with epilepsy Possible connection with acute stress.	Cluster A personality disorder (schizotypal). Close family member(s) with psychotic disorder(s). Obstetric and/or perinatal complications.
Sex	Men < Women	Men < Women	Men > Women
Age	5‐76 (Mean: 23)	15‐50 (Mean: 30)	Men: 18‐25a Women: 25‐35a; >40a
Psychiatric prodromes (onset)	Days‐Weeks Unnoticed (no functional impairment)	Days‐Weeks Unnoticed (no functional impairment)	Months‐years (1‐5a) Functional deterioration
Psychiatric prodromes (phenomenology)	Slight mood swings. Alterations in sleep patterns.	Slight mood swings. Alterations in sleep patterns.	Cognitive symptoms (working memory, verbal memory, and processing speed). Negative symptoms (associability, abulia, anhedonia, affective flattening, and alogia). Diminished (less intense) or brief psychotic symptoms (shorter duration) may appear.
Acute phase (presentation)	Acute (hours‐days), polysymptomatic, fluctuating	Acute (hours‐days), polysymptomatic, fluctuating	Insidious onset, fixed combination of symptoms
Acute phase (phenomenology)	Fluctuating consciousness. Mood swings. Paranoid pan‐anxiety. Particular concern with death. Polymorphic delusional ideas. Altered thought patters (forgetfulness/mutism). Hallucinations in all systems (more typically auditory, but more characteristically visual). Alterations in psychomotricity (hyperkinesia‐akinisia).	Fluctuating consciousness. Mood swings. Paranoid pan‐anxiety. Particular concern with death. Polymorphic delusional ideas. Altered thought patters (forgetfulness/mutism). Hallucinations in all systems (more typically auditory, but more characteristically visual). Alterations in psychomotricity (hyperkinesia‐akinisia).	No alteration in consciousness One or more types of delusional ideas (typically paranoid), generally stable within a single episode. Altered thought patterns. Hallucinations in all systems (most commonly auditory and synesthetic; third‐persons auditory hallucinations that make comments or punish, more characteristically; visual hallucinations less common).
DUP	Days‐ weeks	Days‐ weeks	Mean: 8.4m/ Median: 3m
Antipsychotic intolerance	Frequent	Infrequent	Infrequent, varies by sex of patient
ECT response	Variable, it can be partial or transient	Good	Variable
Evolution	Neurological complications.	Cyclical. Fast resolution of episodes.	Chronic. Progressive resolution of episodes.
Prognosis	Appearance of cognitive interference and decreased impulse control.	Benign in the long term (lesser presence of residual symptoms)	Persistence of negative cognitive symptoms.

Abbreviations: DUP, Duration of Untreated Psychosis; ECT, Electroconvulsive therapy.

In conclusion, we suggest that patients who initially exhibit an atypical psychotic profile should be subjected to blood analysis (and most importantly, to cerebrospinal fluid analysis) to determine whether antibodies against neuronal cell surface or synaptic receptors are present, so as to be able to rule out a possible diagnosis of autoimmune encephalitis. The markers that should raise suspicion of the presence of autoimmune encephalitis in a first episode of psychosis include (a) a lack of long‐term (cognitive and negative) psychiatric prodromes; (b) an atypical psychotic clinical profile; and (c) hypersensitivity to the side effects of antipsychotic medications. These first signs may help arrive at a differential diagnosis. To investigate whether these markers could be useful for detecting anti‐NMDAR encephalitis in its presentation phase, it would be necessary to undertake prospective comparative studies in which patients presenting a first episode of psychosis are duly evaluated (eg analysis of determination of anti‐NMDAR antibodies in the CSF).

## AUTHOR CONTRIBUTIONS

GS: designed, hypothesized, drafted, and revised the manuscript. BQ: designed and drafted the manuscript. GB: revised the manuscript. CR: revised the manuscript. TR: revised the manuscript. NG: revised the manuscript. D: drafted and revised the manuscript. CP: drafted and revised the manuscript.

## CONFLICT OF INTEREST

None declared.
